# Safety, tolerability, and pharmacokinetics of faldaprevir after single increasing doses in healthy subjects

**DOI:** 10.3389/fphar.2025.1622249

**Published:** 2025-09-03

**Authors:** Chan-Loi Yong, Regina Sennewald, Gerhard Nehmiz, Anne-Marie Quinson, Fenglei Huang

**Affiliations:** ^1^ Boehringer Ingelheim Pharmaceuticals, Inc., Ridgefield, CT, United States; ^2^ PHAROS GmbH, Pharmaceutical Research Outsourcing, Ulm, Germany; ^3^ Boehringer Ingelheim Pharma GmbH & Co. KG, Biberach, Germany

**Keywords:** faldaprevir, pharmacokinetics, HCV, NS3/4A, OATP 1B

## Abstract

Faldaprevir (FDV) is a novel NS3/NS4A inhibitor used in the treatment of hepatitis C infection in an interferon-free regimen. This study evaluated the safety, tolerability, and pharmacokinetics of FDV following a single dose in healthy male subjects and assessed the effect of food on FDV bioavailability. In the placebo-controlled, randomized, single-blind, single-increasing-dose part of the study (Part 1), 64 healthy male subjects were randomized to receive FDV in PEG/TRIS/meglumine solution at one of eight dose levels (4–1,200 mg, n = 6 per dose group) or placebo (n = 2 per dose group). In Part 2, the effect of food on the relative bioavailability (rBA) of 480 mg FDV in solution was evaluated in an open-label, crossover comparison, with and without a high-fat breakfast, in an additional 10 subjects (8 FDV and 2 placebo). Following single doses of 4–1,200 mg FDV, geometric mean (gMean) C_max_ and AUC_0-inf_ were 3.57–16500 ng/mL and 254–402000 h*ng/mL, respectively, displaying more than dose-proportional increases in exposure. FDV was slowly absorbed, with gMean t_1/2_ and median t_max_ of 15.5–39.2 h and 4.0–14.0 h, respectively; both were dose dependent. The urinary excretion of FDV was less than 0.1% of the dose. A high-fat breakfast increased systemic exposure to FDV in solution by 14%. FDV was generally well tolerated; subjects who experienced adverse events (AEs) recovered without sequelae, and no serious AEs were reported. Indirect (unconjugated) bilirubin of >3.0 mg/dL was observed in two subjects at 480 mg and five subjects at 1,200 mg. In conclusion, at single doses of 4–1,200 mg in healthy male subjects, FDV showed dose-dependent pharmacokinetics and was generally considered safe and well tolerated. Food had no clinically relevant effect on the rBA of FDV.

## Introduction

Chronic hepatitis C virus (HCV) infection remains a major global health challenge, affecting more than 180 million individuals worldwide and leading to severe liver-related complications, including cirrhosis and hepatocellular carcinoma (Lavanchy, 2009). The advent of direct-acting antiviral agents (DAAs) targeting specific HCV proteins, such as the NS3/4A protease, has revolutionized treatment strategies, particularly for the difficult-to-treat genotype-1 (GT-1).

Faldaprevir (BI 201335) is a potent, selective HCV NS3/4A protease inhibitor. Preclinical evaluations demonstrated faldaprevir’s robust antiviral activity against HCV genotypes (GTs) 1, 4, 5, and 6 (White et al., 2010), along with a favorable absorption, distribution, metabolism, and excretion (ADME) profile in multiple animal models. These studies also highlighted its high metabolic stability and remarkable liver distribution, predicting promising pharmacokinetics (PK) and target organ exposure in humans (White et al., 2010). Faldaprevir (FDV) is a substrate of CYP 3A, a substrate of OATP 1B1, and a substrate and an inhibitor of P-gp (Chen et al., 2014; Joseph et al., 2014; Huang et al., 2017; 2021). At a therapeutic dose of 120 mg QD, FDV shows inhibitory effects against CYP2C9 and weak inhibitory effects against CYP3A4 but has no effect on other CYPs. Moreover, in combination with pegylated interferon alfa-2a (PegIFN) and ribavirin (RBV), faldaprevir achieved sustained virologic response (SVR) rates of up to 84% in treatment-naïve patients, without exacerbating the adverse event profile of the existing treatment regimen (Ferenci et al., 2015). In addition, it was also shown in Phase II trials that faldaprevir could be used in interferon-free regimens in which faldaprevir was combined with an NS5B polymerase inhibitor, deleobuvir (Zeuzem et al., 2014; 2015). FDV is currently under development for an interferon-free regimen for treatment of HCV infection (NCT02593162, 2024; NCT02716428, 2024) and is also considered for the treatment of COVID-19 (Keretsu et al., 2020; Luan et al., 2020; Gammeltoft et al., 2021).

The objective of this study was to assess the safety, tolerability, and pharmacokinetics (including assessment of dose linearity) of faldaprevir after single ascending oral doses and to preliminarily assess the effect of food on the pharmacokinetics of faldaprevir.

## Materials and methods

The study was conducted at the PHAROS GmbH, Ulm, Germany, in accordance with the International Conference on Harmonization guidelines for Good Clinical Practice and the principles of the Declaration of Helsinki. Before study initiation, the clinical trial protocol, the subject information, and the informed consent form were reviewed by the responsible local Independent Ethics Committee (Ethikkommittee der Landesärztekammer Baden-Württemberg, Stuttgart, Germany). The clinical trial application was also reviewed by the German Competent Authority (BfArM, Bonn, Germany).

### Subjects

After signing the written informed consent, healthy male volunteers (aged 18–50 years with a body mass index (BMI) between 18.5 and 29.9 kg/m^2^) were enrolled in the study. Subjects were in generally good health, as determined by medical history, physical examination, and clinical laboratory tests. Exclusion criteria included clinically abnormal laboratory results, evidence of existing diseases or disorders, or any observations or conditions (e.g., smoker of more than 10 cigarettes/day, excessive consuming of alcohols, and drug abuses) which might interfere with the pharmacokinetics of the study drug. Subjects could be withdrawn from the study at any time due to inclusion/exclusion criteria violations, withdrawal of consent, intake of concomitant drugs interfering with the study medication, or other medical reasons [e.g., surgery, adverse event (AEs), or other diseases].

### Study design

**FIGURE 1:**
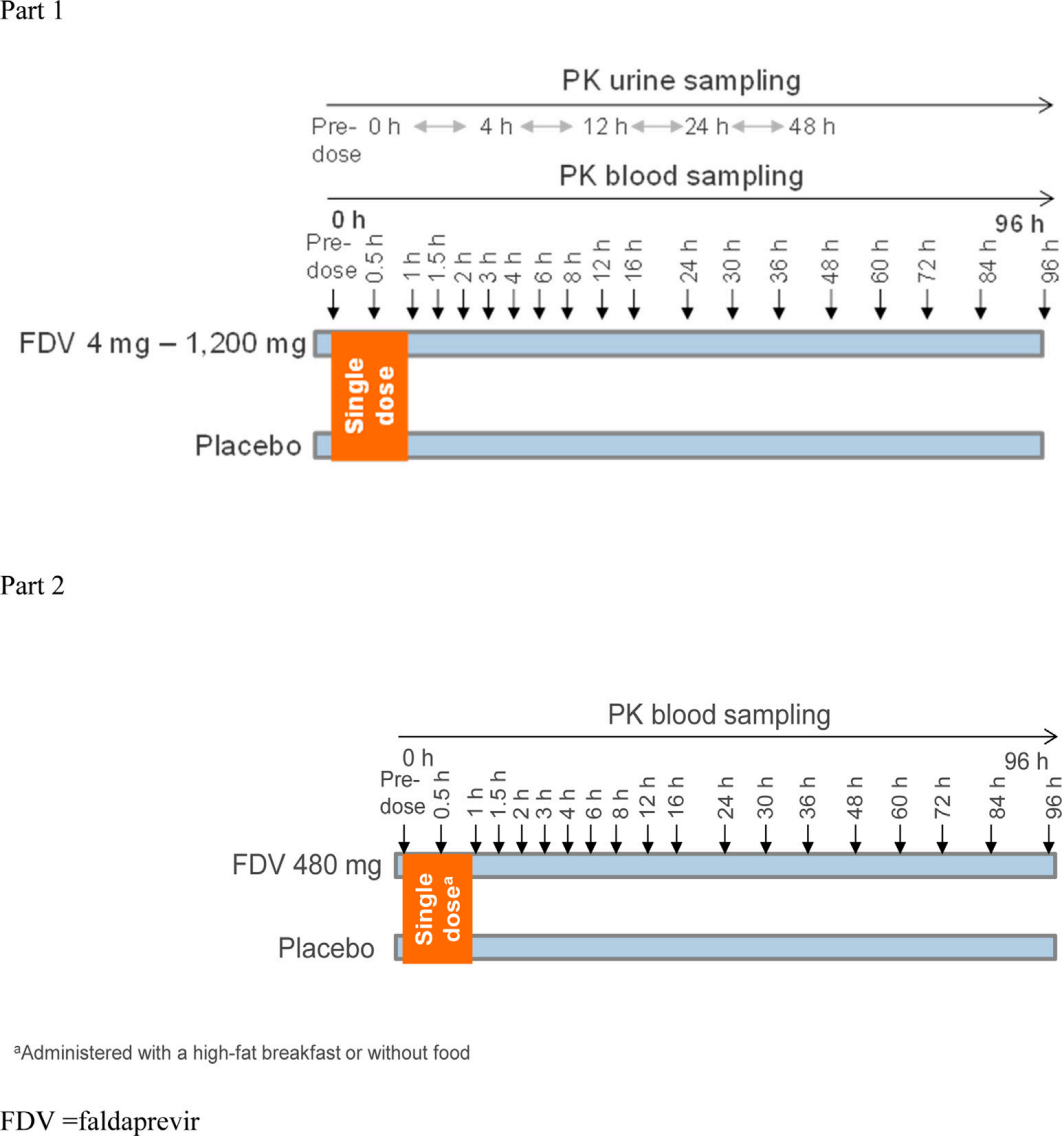
Study design.

Part I of the single-center, randomized, single-blind, placebo-controlled, and single dose-escalation (4, 16, 48, 120, 240, 480, 800, and 1,200 mg) study was designed to evaluate the pharmacokinetics and document the safety and tolerability of FDV in healthy male volunteers (Figure 1). Six volunteers received the active drug at each dose level dissolved in a polyethylene glycol (PEG)/tromethamine (TRIS)/meglumine solution, and two received a placebo. One dose level was tested within each group. Subject groups were dosed and evaluated sequentially, beginning with the lowest-dose group and proceeding stepwise to the highest-dose group. The next higher dose was administered only if no safety concerns arose during treatment of the preceding group. The placebo or FDV PEG/TRIS/meglumine solution was administered with 240 mL of water in the morning of each study day after overnight fasting. Part II of the study, an open-label, randomized, two-period-crossover study in an additional 10 healthy male subjects, was designed to evaluate the effect of food on the relative bioavailability of FDV. The subjects received single-dose FDV 480 mg in PEG400/TRIS/meglumine solution (n = 8) or placebo solution (n = 2) with and without a high-fat breakfast (the composition of breakfast aligned with the FDA Guideline); the subjects were blinded to receive active or placebo.

### Blood and urine sampling

#### Blood sampling

Blood samples were taken pre-dose (0) and at prespecified time points after dosing (Figure 1). At each time point, approximately 4.9 mL of blood was drawn into evacuated collection tubes, containing ethylene diamine tetraacetate (EDTA) anticoagulant and labeled with sample identification information. The EDTA-anticoagulated blood samples were immediately placed on ice and centrifuged at 4 °C and approximately 2000–4,000 g for approximately 10 min in a refrigerated centrifuge as soon as possible after collection. The harvested plasma was split into two aliquots, immediately placed in appropriately labeled tubes, and stored at −20 °C or below until shipment.

#### Urine sampling

A blank urine sample was collected prior to drug administration, and two 10-mL aliquots were retained to be checked for analytical interference. All urine voided during the sampling intervals 0–4, 4–12, 12–24, and 24–48 h after administration was collected in containers (Figure 1). Subjects were asked to completely void their bladder at the end of each collection interval. The urine volume for each collection interval was documented. Two aliquots of approximately 5 mL were taken and frozen at −20 °C or below and stored for bioanalytical measurement. Until shipped on dry ice to the analytical laboratory, the urine samples were stored at −20 °C or below at the clinical site and stored in the analytical laboratory at −20 °C or below until analysis.

### Bioanalytical methods

A validated high-pressure liquid chromatography–tandem mass spectrometry (HPLC-MS/MS) method was used to quantify concentrations of faldaprevir in EDTA plasma (Joseph et al., 2014; Huang et al., 2021). The calibration range was from 0.2 to 250 ng/mL for FDV; the assay accuracy was ≤8.95% and ≤5.38% for intra- and inter-run, respectively; the assay precision (CV%) was ≤7.69% and ≤6.32% for intra- and inter-run, respectively. A validated HPLC-MS/MS method was used to quantify concentrations of faldaprevir in urine. The calibration range was from 1 to 1,000 ng/mL for FDV; the assay accuracy was ≤6.00% and ≤−2.33% for intra- and inter-run, respectively; the assay precision (CV%) was ≤5.82% and ≤5.21% for intra- and inter-run, respectively.

### Safety assessments

Safety was assessed throughout the study by monitoring changes in clinical laboratory parameters (e.g., serum biochemistry, hematology, and urinalysis), 12-lead electrocardiograms, or vital signs (blood pressure, pulse rate, and temperature measurements); monitoring for occurrence of AEs); and carrying out physical examinations.

### Pharmacokinetic analysis

Plasma FDV concentration–time data were analyzed using a non-compartmental approach with WinNonlin™ (version 5.2, Cary, NC) (Huang et al., 2017; Huang et al., 2008a). Standard formulas for non-compartmental pharmacokinetic analysis provided by WinNonlin were used in determination of the maximum concentration (C_max_), time to maximum concentration (t_max_), oral clearance (CL/F), mean residence time (MRTpo), and apparent volume distribution in terminal phase (V_z_/F). The area under the plasma concentration–time curve (AUC) was calculated using a linear up/log down trapezoidal algorithm. The predicted concentration at the last time-point with quantifiable concentration was used for extrapolation of AUC. The fraction excreted as unchanged drug in urine (f_e_) was calculated as the percentage of the amount of drug excreted in urine as unchanged drug (A_et1–t2_) compared to the administered dose (Huang et al., 2008b).

### Statistical analysis

The power model was used for the analysis of dose proportionality in terms of AUC_0_–  ∞ and C_max_. The model is described using the following equation: Y_km_ = α•D_k_^β^ •e_km_. Logarithmic transformation yields the linear regression equation as follows: Ln Y_km_ = ln α + β•ln D_k_ + ln e_km_, where Y_km_ = response (AUC_0_–  ∞ , C_max_) measured on subject m receiving dose k; ln α = the intercept; β = the slope; D_k_ = the *k*th dose effect, k = 1, 2, …; e_km_ = the random error associated with the *m*th subject who received dose k. Dose linearity requires that β equals 1. Food effect was determined using an analysis of variance (ANOVA) with terms for “subject” (random), “period,” and “treatment” (fixed), based on active-treated subjects. Statistical analysis was performed using the SAS^®^ program system (version 8.2, Cary, NC) (Huang et al., 2008b).

## Results

### Subjects

A total of 64 eligible subjects participated in the single ascending dose (SAD) study of FDV. A total of 10 subjects participated in the food effect study. Two subjects from the SAD part were lost to follow-up (one from the placebo group and one from the 800 mg group) and did not complete the planned observation period; however, the safety data were collected for these two subjects. All were Caucasian male subjects; the median age, weight, and height of volunteers were 39 years (range: 19–49), 80 kg (range: 58–100), and 180 cm (range: 166–199), respectively. There were no major differences in demographic and baseline data among the subjects in the different treatment groups (including the placebo group).

### Pharmacokinetics of FDV after single ascending dose and with and without food

**FIGURE 2:**
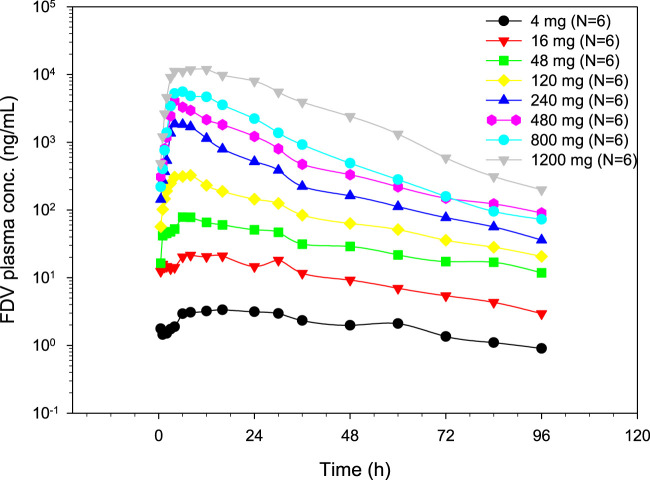
Geometric mean plasma concentration time profiles of FDV after single oral dose administration.

**FIGURE 3:**
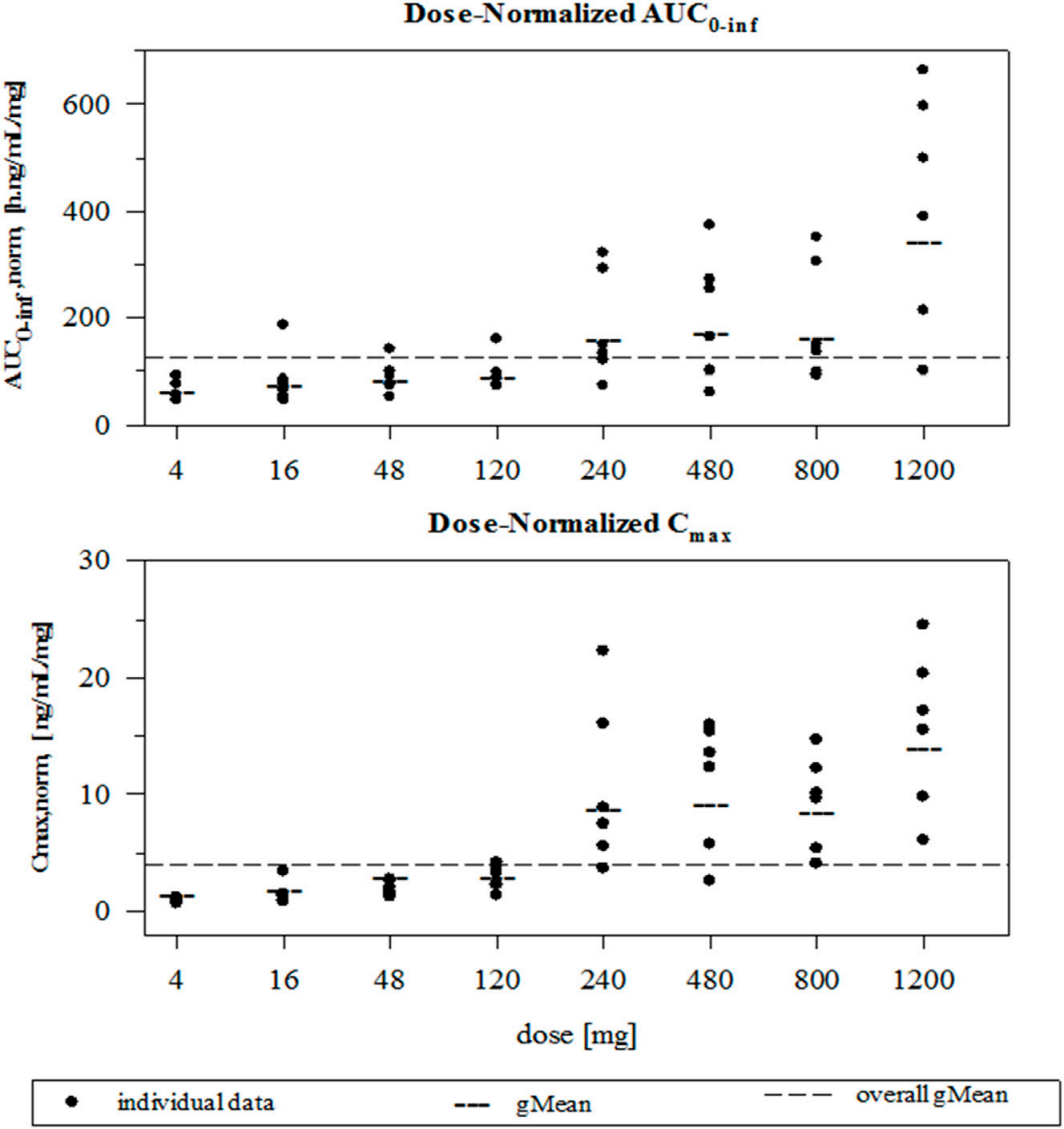
Individual and geometric mean dose normalized AUC_0_–  ∞ and C_max_ values after FDV oral administration DV.

FDV was generally slowly absorbed, especially at low doses, with median t_max_ ranging from 4 to 14 h (Figure 2; Table 1). FDV t_1/2_ seemed prolonged at the lowest doses (approximately 39 h at 4 mg) and shorter at the two highest doses (approximately 16 h at 800 mg and 1,200 mg), but this is possibly a floor effect. Both AUC_0_–  ∞ and C_max_ increased supra-proportionally (Figure 3). For a 10-fold increase in dose, AUC_0_–  ∞ and C_max_ increased by approximately 40- and 50-fold, respectively. Over the whole dose range, the exponent β for the power model for C_max_ was 1.4965 (95% CI: 1.4128–1.5802), indicating a supra-linear increase. No subregions of the dose range could be found where the increase was less steep. For AUC_0_–  ∞ , over the whole dose range, the exponent β for the power model was 1.2532 (95% CI: 1.1703–1.3361), also indicating a supra-linear increase. Again, no sub-regions of the dose range could be found where the increase was less steep. Inter-subject variability was moderately high for the higher-dose groups, with geometric coefficient of variation (gCV) values for AUC_0_–  ∞ in the range of 60–80% compared with approximately 30% for most of the lower-dose groups (Table 1). Urinary excretion of FDV accounted for <0.1% of the administered dose for all dose groups (Table 1).

**TABLE 1:** Pharmacokinetic parameters after single increasing dose of FDV.

Parameter^a^	FDV 4 mg	FDV 16 mg	FDV 48 mg	FDV 120 mg	FDV 240 mg	FDV 480 mg	FDV 800 mg	FDV 1,200 mg
AUC_0–tz_, ng·h/mL	201 (19)	1,030 (52)	3,280 (26)	9,660 (29)	36,100 (60)	74,100 (71)	127,000 (62)	397,000 (83)
AUC_0_– ∞ , ng·h/mL	254 (26)	1,190 (52)	3,980 (34)	10,700 (30)	37,400 (61)	80,700 (77)	128,000 (61)	402,000 (83)
C_max_, ng/mL	3.6 (21)	22.8 (49)	86.6 (33)	344 (46)	2,070 (75)	4,300 (83)	6,630 (53)	16,500 (55)
t_max_, h	14 (8–24)	8 (6–30)	8 (6–16)	7 (4–8)	4 (3–8)	4 (4–6)	9 (4–12)	8 (4–24)
t_½_, h	39.2 (29)	31.8 (27)	37.7 (28)	27.4 (28)	22.2 (14)	33.3 (55)	16.1 (17)	15.5 (26)
CL/F, mL/min	256 (26)	218 (52)	196 (34)	183 (30)	104 (61)	96.7 (77)	101 (61)	48.5 (83)
Vz/F, L	868 (15)	600 (55)	640 (16)	435 (29)	200 (55)	278 (84)	141 (72)	65 (119)
Fe_0–48h_, %^b^	NC	NC	0.01 (165)	0.02 (93)	0.04 (68)	0.04 (37)	0.03 (110)	0.03 (54)

^a^ All parameters are presented as geometric mean (gCV%) except t_max_, which is presented as median (range).

^b^ Fraction excreted in urine.

FDV, faldaprevir; NC, not calculated.

Median t_max_ occurred at 6 h under fasted conditions and at 4 h under fed conditions. The AUC_0_–  ∞ and C_max_ increased by 14% and 9%, respectively, after a high-fat and high-calorie breakfast. The upper boundaries of the 90% confidence intervals (CIs) for both C_max_ and AUC_0_–  ∞ were outside the prespecified no-effect value of 125%, suggesting that FDV bioavailability increased slightly with food intake (Table 2).

**TABLE 2:** Pharmacokinetic parameters of 480 mg FDV under fasted and fed conditions.

Parameter^a^ N = 7^b^	FDV 480 mg/fasted	FDV 480 mg/fed	Ratio (fed:fasted)^c^	90% CI for ratio^c^
AUC_0_– ∞ , ng·h/mL	101,775	89,620	113.6	92.7, 139.1
C_max_, ng/mL	5,603	5,145	108.9	82.3, 144.1
AUC_0–tz_, ng·h/mL	83,400 (23)	92,043 (12)	NC	NC
t_max_, h	4 (4–6)	6 (4–12)	NC	NC
t_½_, h	19.6 (18)	18.0 (23)	NC	NC
CL/F, mL/min	98 (34)	85 (14)	NC	NC
Vz/F, L	161 (34)	132 (27)	NC	NC

^a^ All parameters are presented as mean (CV%) except AUC_0_–  ∞ and C_max_, which are presented as adjusted gMean, and t_max_, which is presented as median (range).

^b^ Excluding data from one subject who vomited 24 min after dose without food and exhibited highest exposure with food.

^c^ Presented as %.

FDV, faldaprevir; NC, not calculated.

### Safety

FDV administered as an oral solution was generally well tolerated. All subjects who experienced an AE recovered without sequelae. No serious AEs occurred during the study. The most frequently observed AEs were gastrointestinal disorders for both parts. No clinically relevant changes in vital signs, ECGs, or medical examinations were observed. The AE profiles for part 1 are summarized in Table 3. A total of 26 subjects out of 48 had an AE after treatment with FDV, and 4 subjects out of 16 in the placebo group reported an AE. Adverse events with a possible relationship to the study drug according to the investigator were observed in 19 subjects receiving FDV. Most AEs are mild or moderate in intensity; one subject (FDV 240-mg dose group) reported severe vomiting. For part 2, AEs were similar between the fasted and fed state. Six of the 10 subjects reported AEs of mild or moderate intensity. Diarrhea and nausea were experienced by three subjects in FDV and one subject receiving placebo; vomiting was experienced by two subjects in FDV and one subject receiving placebo; headache was experienced by one subject in FDV and one subject receiving placebo.

**TABLE 3:** Summary of the most frequently reported adverse events with treatment.

n (%)	Placebo	FDV 4 mg	FDV 16 mg	FDV 48 mg	FDV 120 mg	FDV 240 mg	FDV 480 mg	FDV 800 mg	FDV 1,200 mg
Number of subjects	16 (100)	6 (100)	6 (100)	6 (100)	6 (100)	6 (100)	6 (100)	6 (100)	6 (100)
Subjects with any AE	4 (25.0)	1 (16.7)	0	3 (50.0)	1 (16.7)	5 (83.3)	6 (100)	4 (66.7)	6 (100)
AEs by system organ class^a^ Preferred term									
Gastrointestinal disorders	2 (12.5)	1 (16.7)	0	0	1 (16.7)	2 (33.3)	4 (66.7)	4 (66.7)	4 (66.7)
Nausea	1 (6.3)	1 (16.7)	0	0	0	1 (16.7)	2 (33.3)	1 (16.7)	1 (16.7)
Vomiting	0	0	0	0	0	1 (16.7)	1 (16.7)	0	0
Retching	0	0	0	0	0	0	2 (33.3)	0	1 (16.7)
Flatulence	0	0	0	0	0	0	1 (16.7)	0	1 (16.7)
Diarrhea	1 (6.3)	0	0	0	1 (16.7)	0	3 (50.0)	4 (66.7)	3 (50.0)
Abdominal pain	0	0	0	0	0	1 (16.7)	0	1 (16.7)	0
Hepatobiliary disorders	0	0	0	0	0	0	2 (33.3)	0	5 (83.3)
Hyperbilirubinemia	0	0	0	0	0	0	2 (33.3)	0	5 (83.3)
Jaundice	0	0	0	0	0	0	0	0	1 (16.7)^b^
Nervous system disorders	4 (25.0)	1 (16.7)	0	1 (16.7)	0	2 (33.3)	4 (66.7)	1 (16.7)	1 (16.7)
Headache	4 (25.0)	1 (16.7)	0	1 (16.7)	0	2 (33.3)	3 (50.0)	1 (16.7)	0
Dizziness	0	0	0	1 (16.7)	0	0	1 (16.7)	1 (16.7)	1 (16.7)

^a^ More than one AE can occur in a single subject.

^b^ Subject developed mild transient jaundice of the skin and sclera.

FDV, faldaprevir.

Marked hyperbilirubinemia (>3.0 mg/dL) was observed in seven subjects in Part 1: two subjects in the 480-mg group and five subjects in the 1,200-mg group. Increases in total bilirubin were observed in a dose-dependent manner: two subjects in 120 mg, three subjects in 240 mg, five subjects in 480 mg, six subjects in 800 mg, and six subjects in 1,200 mg dose groups in Part 1; two subjects receiving placebo; eight subjects who received 480 mg in Part 2. All these were associated with elevations of indirect bilirubin. Slight elevations of direct bilirubin were observed in 14 subjects. For those with hyperbilirubinemia or with elevated total bilirubin levels, no clinically relevant changes in alanine transaminase, hemoglobin, or neutrophil count were observed.

## Discussion

The objectives of this study were 1) to characterize the pharmacokinetics of FDV, an inhibitor of HCV NS3/4A, in healthy male volunteers after single ascending oral-dose administration, 2) to preliminarily evaluate the effect of food on the pharmacokinetics of FDV, and 3) to document its safety and tolerability in these subjects.

For the SRD part of the study, six subjects received active drugs and two received placebos. Due to the lack of prior clinical data, it is usually not possible to accurately determine the sample size for the first time in human studies; however, it is generally believed that the selected sample size (6 + 2) is sufficient for the evaluation of the safety and preliminary pharmacokinetics of the new chemical entity (NCE) (Broom, 1990; Huang et al., 2008b).

This study was a first-in-human trial conducted in healthy male volunteers. However, the pharmacokinetics and safety of FDV in special populations, including HCV-infected patients and those with liver dysfunction, were evaluated in subsequent clinical studies, with findings reported in the previously published literature (Huang et al., 2016; Ferenci et al., 2015).

FDV has been shown to be actively absorbed into the liver. In rats, FDV demonstrated a 42-fold enrichment in the liver compared with plasma (Duan et al., 2012), which was accurately predicted using a rat HepatoPac model (Ramsden et al., 2014a). Using a human Hepato Pac model, 22–32-fold enrichments in the liver was predicted in humans (Ramsden et al., 2014b). Three processes contributed approximately equally to the total absorption of FDV, namely; (i) active uptake inhibited by rifamycin SV (related to OATP); (ii) active uptake not inhibited by rifamycin SV; (iii) and passive permeability (Li et al., 2014). With increased dose and concentration of FDV, the active hepatic uptake of FDV may be saturated and lead to more than a dose-proportional increase in plasma FDV concentrations. This may have contributed to a supra-proportional increase in AUC and C_max_ observed in the current study. The saturation of uptake of FDV into the liver may result in a concomitant decrease in the volume of distribution and systemic clearance, and it may lead to a minimal impact on the plasma t_1/2_. The observed relatively longer t_1/2_ at lower dose (≤120 mg) and relatively similar t_1/2_ at higher dose [≥240 mg; considering t_1/2_ values of 19.6 (CV% = 18%) for 480 mg from the food effect part suggested that, at low doses, FDV may be distributed (uptake) to tissues (compartment) other than the liver or at higher dose, FDV distributed far less to other tissues. A similar pattern of nonlinear pharmacokinetics was observed for grazoprevir (ZEPATIER™, 2024) and simeprevir (OLYSIO^®^, 2024); both are NS3/4A HCV protease inhibitor, with hepatic uptake mediated by OATP1B. For simeprevir, a PBPK model suggested that saturation of OATP1B played a major role in the contribution of the observed non-linearity (Snoeys et al., 2016). For grazoprevir, the FDA clinical pharmacology reviewer agreed with the sponsor’s hypotheses that saturation of the OATP1B-mediated uptake process contributed to nonlinear PK observed for grazoprevir (FDA, 2012). The observed saturation of hepatic uptake via OATP1B as a potential cause of nonlinear pharmacokinetics for faldaprevir warrants further investigation. *In vitro* transporter studies and PBPK modeling could help validate this hypothesis and are considered potential directions for future research.

FDV is metabolized by CYP3A4 and is a substrate of the efflux transporter of P-gp; thus, saturation of these two processes may also contribute to the nonlinear PK observed in the present study. However, since systemic CYP3A4 (in the liver), but not intestinal CYP3A4, contributed to most of the metabolism of FDV (Li et al., 2014), and the half-life of FDV was not prolonged with the increased dose in the current study, saturation of CYP3A4 metabolism was not considered the main cause of the observed non-liner PK of FDV. Given the km values of 11.9 µM, at the dose of 120 mg (assuming intestinal volume of 250 mL), the efflux processes mediated by P-gp administration would be saturated already (Wu et al., 2016); thus, the contribution of the saturation of P-gp to the observed nonlinear PK of FDV was probably limited.

A clinically irrelevant increase in bioavailability by food was observed in the current study. This appeared to be consistent with the fact that FDV is a BCS II compound as it has been shown that food may increase the exposures of BCS II compounds (Lentz, 2008). The observed slight increase in BA under fed conditions in the current pilot study of FDV in solution was in good agreement with the observed increased BA when FDV capsules were given with high-fat and high-calorific breakfast in the later pivotal final food effect study (Wu et al., 2016). The 90% CI exceeding 125% may be attributable to underpowering as this is a pilot food-effect study. The slight increase in FDV exposure under fed conditions is not considered clinically relevant. In the FDV clinical development program, both 120-mg and 240-mg doses were evaluated in Phase II/III trials and demonstrated comparable safety profiles (Ferenci et al., 2015), with 120 mg selected as the therapeutic dose. FDV exposure at 240 mg was approximately 5-fold higher based on trough concentrations (Huang et al., 2016) and up to 7-fold higher based on AUC_0–24_,_ss_ (Huang et al., 2021) compared to the 120-mg dose in HCV-infected patients. Therefore, the modest increase in exposure observed in the fed state falls within the established safety margin, and no dose adjustment is required when FDV is co-administered with high-fat or high-calorie food.

AEs observed in this study were consistent with the known FDV safety profiles (Chen et al., 2014; Huang et al., 2014; 2017; 2021; Joseph et al., 2014; 2015; Wu et al., 2016). Elevation of unconjugated bilirubin was observed in several subjects in the present study. It is likely related to the interaction of FDV with bilirubin. Bilirubin, formed from hemoglobin degradation, binds to albumin in the circulation, dissociates in hepatic blood flow, and enters hepatocytes via OATPs. Once inside hepatocytes, bilirubin binds to cytoplasmic proteins and is conjugated to glucuronic acid by UGTs in the endoplasmic reticulum. This conjugation process helps enhance bilirubin’s water solubility and thus its intrinsic clearance. Glucuronidated bilirubin is actively transported into bile via MRP2 at the canalicular membrane, and then, it is cleared into the feces (Sticova and Jirsa, 2013; Tátrai and Krajcsi, 2020). FDV is an inhibitor of OATP1B1, OATP1B3, UGT1A1, and MRP2, so FDV inhibits all steps of *in vivo* disposition of bilirubin and thus leads to benign accumulation of unconjugated bilirubin (Joseph et al., 2014; Wu et al., 2016; Huang et al., 2017; 2021); When FDV-mediated UGT inhibition is combined with predisposing genetics causing impaired bilirubin clearance, the incidence of benign hyperbilirubinemia (indirect bilirubin of >3.0 mg/dL) increases. In fact, genotyping in the seven subjects with an elevation of indirect bilirubin of >3.0 mg/dL in the current study showed a polymorphism (UGT1A1*28) associated with benign indirect hyperbilirubinemia in Gilbert’s Syndrome (Rotger et al., 2005; Lankisch et al., 2006) in six of these subjects. For all subjects with an elevation of benign unconjugated bilirubin, no clinically relevant changes in alanine transaminase, hemoglobin, or neutrophil count were observed. Slight elevations in direct bilirubin were observed in 14 subjects, and it is likely due to methodical overestimation of direct bilirubin via the Diazo reaction (Doumas and Wu, 1991; Kazmierczak et al., 2002). After comprehensively analyzed *in vitro*, preclinical and clinical data on the FDV development program, it was concluded that the FDV-mediated hyperbilirubinemia is not associated with any liver injury or toxicity and is considered to result from decreased bilirubin elimination due to the drug–bilirubin interaction (Sane et al., 2014). Elevated unconjugated bilirubin (hyperbilirubinemia) was also observed in patients treated with atazanavir (an inhibitor of UGTs and OATPs) and simeprevir (inhibitor of OATPs). Due to the benign nature of this observation (resulted from the interaction disposition process and reversible upon discontinuation), the observed hyperbilirubinemia is considered not important as it is not associated with any liver injury (Lankisch et al., 2006; OLYSIO^®^, 2024). In clinical studies involving faldaprevir, bilirubin levels were closely monitored, and adverse events related to bilirubin elevation were reported and managed following regulatory guidelines. These findings have been documented in previously published studies (Sulkowski et al., 2013; Ferenci et al., 2015; Jensen et al., 2016; Chen et al., 2014; Huang et al., 2014; 2017; 2021; Joseph et al., 2014; 2015; Sane et al., 2014; Wu et al., 2016).

In summary, both AUC_0_–  ∞ and C_max_ increased supra-proportionally to dose. Urinary excretion (f_e_) of FDV was negligible. Food had no clinically relevant effect on FDV exposure (AUC and C_max_). The FDV solution given as single doses of 4–1,200 mg was generally well tolerated: AEs were consistent with the known FDV safety profile; tolerability was reasonable, with increases in gastrointestinal symptoms and indirect hyperbilirubinemia at higher doses; increases in indirect bilirubin concentrations were reversible and not associated with decreases in alanine transaminase, hemoglobin, or neutrophil count. In conclusion, at single doses of 4–1,200 mg in healthy male subjects, FDV showed dose-dependent pharmacokinetics and was generally safe and well tolerated.

## Data Availability

The original contributions presented in the study are included in the article/supplementary material, further inquiries can be directed to the corresponding author.
